# Urinary tract infection in small children: the evolution of renal damage over time

**DOI:** 10.1007/s00467-017-3705-5

**Published:** 2017-07-05

**Authors:** Svante Swerkersson, Ulf Jodal, Rune Sixt, Eira Stokland, Sverker Hansson

**Affiliations:** 1 Department of Pediatric Uronephrologic Center, Sahlgrenska Academy, The Queen Silvia Children’s Hospital, Sahlgrenska University Hospital, 416 85 Göteborg, Sweden; 2 Department of Pediatric Clinical Physiology, Sahlgrenska Academy, The Queen Silvia Children’s Hospital, Sahlgrenska University Hospital, Göteborg, Sweden; 3 Department of Pediatric Radiology, Sahlgrenska Academy, The Queen Silvia Children’s Hospital, Sahlgrenska University Hospital, Göteborg, Sweden

**Keywords:** Urinary tract infection, Children, Vesicoureteral reflux, Renal damage

## Abstract

**Background:**

Our objective was to analyze the evolution of kidney damage over time in small children with urinary tract infection (UTI) and factors associated with progression of renal damage.

**Methods:**

From a cohort of 1003 children <2 years of age with first-time UTI, a retrospective analysis of 103 children was done. Children were selected because of renal damage at index ^99m^Tc-dimercaptosuccinic acid (DMSA) scintigraphy at least 3 months after UTI, and a late DMSA scan was performed after at least 2 years. Damage was classified as progression when there was a decline in differential renal function (DRF) by ≥4%, as regression when there was complete or partial resolution of uptake defects.

**Results:**

Of 103 children, 20 showed progression, 20 regression, and 63 remained unchanged. There were no differences between groups regarding gender or age. In the progression group, 16/20 (80%) children had vesicoureteral reflux (VUR) grade III–V and 13 (65%) had recurrent UTI. In multivariable regression analysis, both VUR grade III–V and recurrent UTI were associated with progression. In the regression group, 16/20 (80%) had no VUR or grade I–II, and two (10%) had recurrent UTI.

**Conclusions:**

Most small children with febrile UTI do not develop renal damage and if they do the majority remain unchanged or regress over time. However, up to one-fifth of children with renal damage diagnosed after UTI are at risk of renal deterioration. These children are characterized by the presence of VUR grades III–V and recurrent febrile UTI and may benefit from follow-up.

## Introduction

Urinary tract infection (UTI) in children can be associated with renal damage. The frequency of renal damage found at ^99m^Tc-dimercaptosuccinic acid (DMSA) scintigraphy 5 months to 2 years after UTI is around 20%, but the proportion varies between different studies [1]. The risk of renal damage may be increased in children with malformations, high-grade vesicoureteral reflux (VUR), and recurrent febrile UTI [2–6]. Moreover, there seems to be a gender variation, where boys more often have congenital renal damage associated with VUR, whereas in girls acquired focal damage related to recurrent UTI is more common [7, 8]. Infections may cause transient inflammatory changes in the kidney that resolve within 3–6 months [9–11].

Studies of long-term consequences of renal damage have shown varying results. While some earlier studies found increased frequency of hypertension, impaired renal function, and pregnancy-related complications, a comprehensive meta-analysis of conducted studies could not confirm these results [12]. However, a study of 86 women with 35 years of follow-up found significant decrease in kidney function and increased frequency of hypertension in those with bilateral and severe renal scarring [13, 14]. Despite many studies concerning different aspects of kidney damage, few have analyzed the evolution of damage over time and the conclusions have been diverging [15–18].

The aim of this study was to describe the evolution of kidney damage in small children with UTI and to analyze possible factors associated with progression. The focus has been on permanent renal damage that persisted beyond 3 months of the index UTI.

## Methods

This study is a retrospective analysis of small children with kidney damage found on DMSA scintigraphy performed at least 90 days after their first symptomatic UTI, here referred to as index DMSA, and who all had a follow-up DMSA scan more than 2 years after the index UTI. They all belonged to a population-based cohort of 1003 children below 2 years of age diagnosed with a first-time symptomatic UTI at the emergency room of the Queen Silvia Children’s Hospital, Göteborg, from 1994 through 2003. Excluded were children with asymptomatic bacteriuria, urinary tract obstruction, urogenital malformation, neurogenic bladder, and severe neurological or systemic disease. The UTI investigation protocol recommended ultrasound, voiding cystourethrography (VCUG), and DMSA scan.

Clinical and laboratory parameters at the index infection, including highest measured temperature, highest C-reactive protein (CRP), bacterial findings, and the number of febrile recurrences, were recorded. The diagnosis of UTI required bacteriuria of a single species of at least 100,000 colony-forming units (CFU)/ml in two midstream or bag samples, 10,000 CFU/ml or more in one catheter sample or any bacterial growth in urine obtained by suprapubic aspiration. Febrile recurrence was defined as UTI with temperature of 38.5 °C or more. The VCUGs were revaluated by the same pediatric radiologist (ES) and the DMSA scans by the same nuclear medicine specialist (RS). VUR was classified according to the International Reflux Study in Children [19]. In case of bilateral VUR, the highest grade was used to classify each case.

DMSA scan was performed according to European guidelines [20]. Kidneys were classified as normal if differential renal function (DRF) was 45% or more and if there was no up-take defect, as a minor defect if DRF was 45% or more with one or more up-take defects, as a moderate defect if DRF was 40% to 45%, and as a pronounced defect if DRF below 40%. If there were bilateral defects or renal duplication, arbitrary classification was done to the same categories. The evolution of the kidney damage was classified into three groups: (1) progression if ≥4% decline of DRF between the index and the last DMSA scan, (2) regression if isotope up-take defects, diagnosed by visual evaluation, on the index DMSA scan were partially or completely resolved at follow-up, (3) unchanged in the remaining cases.

The median time between the index UTI and the index DMSA scan was 8 months (range, 3–61 months) and between the index UTI and the last follow-up DMSA scan 96 months (range, 26–210 months). In 37 children, the index DMSA scan was performed between 3 and 6 months after the index UTI. Of these, seven were classified as regression, 22 as unchanged, and eight as progression at the last follow-up DMSA scan. Corresponding figures when index DMSA scan was performed more than 6 months after UTI showed similar proportions (13, 41, and 12, respectively, *p* = 0.74).

## Statistics

Distribution of continuous variables is given as median, minimum, and maximum and categorical variables as number and percentage. For comparison between the three groups of evolution of kidney damage, the Mantel–Haenszel Chi-square test was used for dichotomous and ordered variables and the Spearman correlation test for continuous and ordered variables. In the assessment of factors associated with progression of kidney damage, univariable analysis was done by logistic regression and all significant univariable variables were entered into a multivariable stepwise logistic analysis. All significance tests were two-sided and conducted at the 5% significance level. The statistical analyses were performed using SAS® software version 9.3.

## Results

**Fig. 1:**
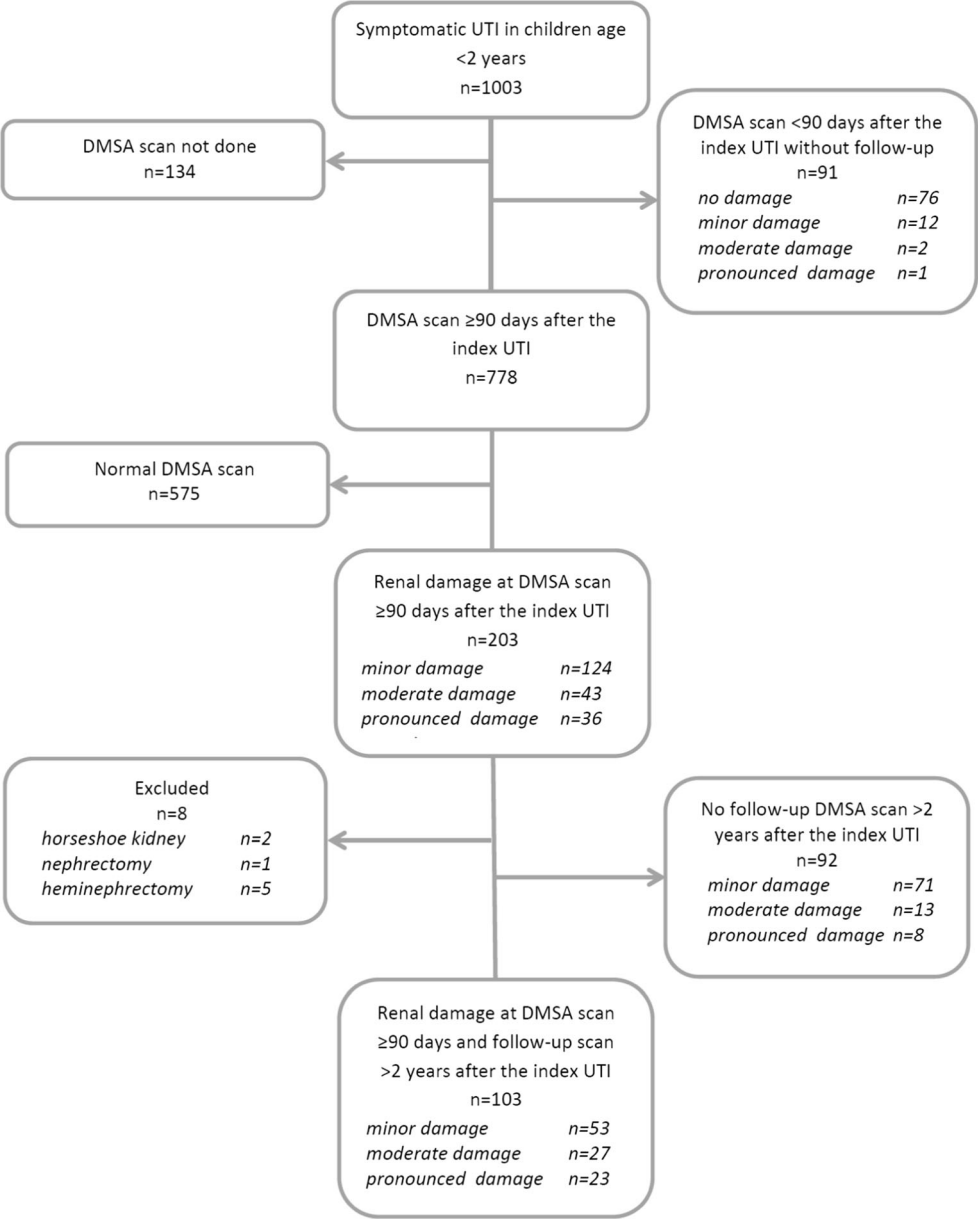
Flow chart of included patients.* DMSA* dimercaptosuccinic acid,* UTI* urinary tract infection

The original cohort consisted of 1003 children (Fig. 1). In 778, the DMSA scan was performed 90 days or more after the index UTI. In 575 of these 778 children, the DMSA scan was normal and not repeated. In 203 children (26%), the DMSA scan showed damage. In comparison with children with a normal DMSA scan, the age and gender distribution were similar, while non-*E.coli* infection, recurrent UTI, and high-grade VUR were more prevalent in the group with renal damage (Table 1). The kidney damage was classified as minor in 124 (61%) children.

**Table 1:** Clinical data of children with and without renal damage at DMSA scan performed ≥90 days after the index UTI

	Without damage (*n* = 575)	With damage (*n* = 203)	*p* value
Gender			
Boys	286	92	0.29
Girls	289	111	
Index UTI			
Age, months,			
median (range)	5.9 (0.2–23.9)	6.3 (0.2–23.6)	0.53
CRP highest, mg/l median (range)	62 (5–350)	120 (5–430)	<0.0001
Bacterial species			
*E. coli*,* n* (%)	542 (94%)	178 (88%)	0.0046
Other Gram-negative sp.	28	16	
Other sp.	5	9	
Recurrent UTI,* n* (%)	65 (11%)	43 (21%)	0.0005
VUR,* n* (%)			
No VUR	484 (88%)	111 (56%)	<0.0001
Grade I	22 (4%)	9 (5%)	
Grade II	28 (5%)	18 (9%)	
Grade III	15 (3%)	25 (13%)	
Grade IV	4 (1%)	31 (16%)	
Grade V	0	4 (2%)	
VCUG not done	22	5	
Renal damage,* n* (%)			
Minor		124 (61%)	
Moderate		43 (21%)	
Pronounced		36 (18%)	

*DMSA* dimercaptosuccinic acid,* UTI* urinary tract infection,* CRP* C-reactive protein,* sp.* species,* VUR* vesicoureteral reflux,* VCUG* voiding cystourethrography

Of the 203 children with renal damage, 92 did not have a follow-up DMSA after 2 years (minor damage in 77%) (Fig. 1). Because of difficulty in interpreting progression of kidney damage, eight children were excluded (two with of horseshoe kidney, one with nephrectomy, and five with heminephrectomy). Thus, 103 children were included, 46 boys and 57 girls, with persistent renal damage 90 days or more after the index UTI and who also had a follow-up DMSA scan after a minimum of 2 years. The median age at the index UTI was 3 months (range, 0.3–17 months) for boys and 8 months (range, 0.7–22 months) for girls. The renal damage was minor in 53, moderate in 27 and pronounced in 23 at index DMSA scan. There was a significant gender difference. Among boys, minor damage was found in 20 (44%), moderate in ten (22%), and pronounced in 16 (35%), while corresponding figures for girls were 33 (58%), 17 (30%), and seven (12%), respectively (*p* = 0.02). Of the 100 children investigated with VCUG, 38 had no VUR, 14 had grade I–II, and 48 had grade III–V. Recurrent febrile UTI occurred in 34 children, 13 boys and 21 girls.

The clinical data for the three groups concerning evolution of kidney damage are given in Table 2. There were no differences between the groups regarding gender, age, highest temperature, or highest CRP at the index UTI.

**Table 2:** Comparison between the three groups of renal damage evolution concerning clinical parameters at the index UTI, number of recurrent UTI, presence of duplex, renal damage at the index DSMA scan, and status of vesicoureteral reflux

	Progression (*n* = 20)	Regression (*n* = 20)	Unchanged (*n* = 63)	*p* value
Gender, boys* n* (%)	10 (50%)	9 (45%)	27 (43%)	0.75
Index UTI				
Age, months median (range)	5.5 (1.3–16.0)	4.6 (0.5–22.0)	6.5 (0.3–21.4)	0.70
CRP, highest, mg/median (range)	135 (23–430)	110 (5–210)	120 (5–300)	0.32
Bacterial species				
*E.coli*,* n* (%)	12 (60%)	18 (90%)	55 (87%)	0.013
Non-*E.coli* sp.	8	2	8	
Recurrent febrile UTI,* n* (%)	13 (65%)	2 (10%)	19 (30%)	<0.001
Renal damage at index DMSA scan,* n* (%)				
Minor	5 (25%)	11 (55%)	37 (59%)	0.048
Moderate	9 (45%)	7 (35%)	11 (17%)	
Pronounced	6 (30%)	2 (10%)	15 (24%)	
VUR,* n* (%)				
no VUR	1 (5%)	14 (70%)	23 (37%)	<0.001
VUR grade I to II	3 (15%)	2 (10%)	9 (14%)	
VUR grade III to V	16 (80%)	3 (15%)	29 (46%)	
VCUG not done		1	2	

*UTI* urinary tract infection,* CRP* C-reactive protein,* sp.* species,* DMSA* dimercaptosuccinic acid,* VUR* vesicoureteral reflux,* VCUG* voiding cystourethrography

The regression group comprised 20 children, nine boys and 11 girls. Ten children had complete resolution of isotope up-take defects. Of those, eight had minor and two moderate damage at the index DMSA scan. One child had VUR grade II, while the others had no VUR. Among the ten children with partial resolution, three had minor, five moderate, and three pronounced damage, and one child had VUR grade II, one grade III, and two grade IV.

In the majority (61%) of children with kidney damage at the index DMSA scan, renal status remained unchanged over time.

## Risk of progression

**Fig. 2:**
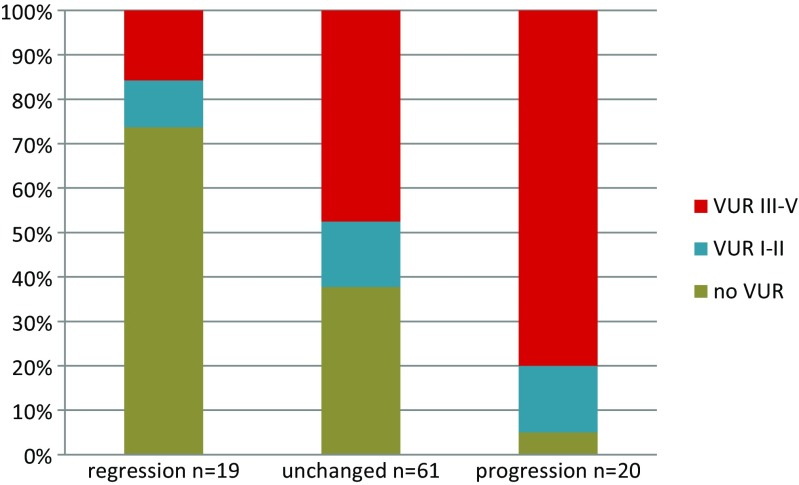
Grade of vesicoureteral reflux related to evolution of renal damage.* VUR* vesicoureteral reflux

The progression group comprised 20 children, ten boys and ten girls. This group was compared with the pooled unchanged and regression groups in univariable analyses (Table 3). Children with moderate or pronounced renal damage at index DMSA scan had a significantly higher risk of progression, 15 of 50 (30%), than children with minor damage, 5 of 53 (9%)(*p* = 0.01). Also, children with VUR grade III–V had a higher risk of progression, 16 of 48 (33%), than children with no VUR or VUR I–II, 4 of 52 (8%) (*p* = 0.002)(Fig. 2).

**Table 3:** Univariable logistic and multivariable stepwise logistic regression analysis of probable explaining factors for progression of kidney damage*.* In the analyses, the progression group is compared with the combined groups of regression and unchanged damage

	Univariable analysis		Multivariable analysis*	
	Odds ratio (95% CI)	*p* value	Odds ratio (95% CI)	*p* value
Bacteriology at the index UTI				
*E. coli* (reference)	1.0			
Non-*E.coli*	4.9 (1.6–14.8)	0.005		
Renal damage at index DMSA scan				
Minor (reference)	1.0			
Moderate or pronounced	4.11 (1.4–12.4)	0.012		
VUR				
Grade 0–II (reference)	1.0			
Grade III–V	6.0 (1.8–19.6)	0.003	4.5 (1.3–15.3)	0.011
Recurrent UTI				
No (reference)	1.0			
Yes	5.5 (1.9–15.6)	0.001	3.8 (1.3–11.5)	0.001

*Area under the ROC curve was 0.77 with the variables in the multivariable model.

*CI* confidence interval,* UTI* urinary tract infection,* DMSA* dimercaptosuccinic acid,* VUR* vesicoureteral reflux

Progression was significantly related to recurrence of UTI, 13 of 34 (38%) compared to 7 of 69 (10%) without recurrence (*p* = 0.001). Recurrence was also significantly related to grade of VUR; in children without VUR, four of 38 (11%) had recurrence, in VUR I–II 7 of 14 (50%) and in VUR III–V 23 of 48 (48%) (*p* < 0.001). Antibacterial prophylaxis was given to 43 of the children with VUR grade III–V with a median duration of 20 months.

In multivariable logistic regression analysis, both VUR grade III–V and recurrent febrile UTI were significantly associated with progression of kidney damage (*p* = 0.011 and *p* = 0.001, respectively) (Table 3). When boys and girls were analyzed separately, there was a significant association only for VUR grade III–V in boys, odds ratio 10.7 (95% CI 1.2–93.6) (*p* = 0.013), while for girls there was a significant association with both recurrent febrile UTI, odds ratio 9.9 (95% CI 1.7–58.1) (*p* = 0.003) and moderate to pronounced damage at the index DMSA scan, odds ratio 7.3 (95% CI 1.2–43.6) (*p* = 0.019).

In the progression group, the mean decrease of DRF in the damaged kidney between the index and the last DMSA scan was 6% (range, 4–10%).

## Discussion

Few studies have investigated the progress of renal damage over time by repeated DMSA scans. In the European branch of the International Reflux Study in Children, Piepsz et al. analyzed the results of serial DMSA scans. A decrease of ≥4% of DFR was chosen as deterioration as it represented one standard deviation at ^99m^Tc-mercaptoacetyltriglycine scintigraphy [15, 21]. Five-year follow-up was performed in 287 children, all with VUR grade III–IV treated with either prophylaxis or surgery. A reduction of ≥4% of DRF occurred in 31 (11%) children, while improved scintigraphic image was seen in eight (3%). Deterioration was more frequent in bilateral VUR grade IV compared to unilateral grade IV or grade III VUR, in children with febrile recurrence and in children under 2 years of age. In a study by Sjöström et al., 108 children with VUR grade III–V were followed for 5 years by repeated renal scintigraphy [17]. A deteriorated DRF of >6% was seen in 18%, while 5% showed recovery of focal lesions. Predictive factors for deterioration were prenatal diagnosis, reduced glomerular filtration rate from the start, breakthrough infections, and grade IV to V VUR. Furthermore, in the Swedish Reflux Trial, 203 children with VUR grade III–IV randomized to prophylaxis, endoscopic surgery, or surveillance were followed for 2 years [16]. New up-take defects or ≥4% decrease in DRF was observed in 24 (12%) children, of whom 15 had recurrent UTI. The patients in these studies were all selected because of high-grade VUR.

In a prospective study by Parvex et al., 50 children 0 to 18 years presenting with DMSA scan defect at 6 months after the first episode of acute pyelonephritis were followed for 3 years [18]. The aim was to analyze the progression of renal scarring over time and its impact on renal growth. In total, 88 renal units showed focal scars on DMSA scan at 6 months. Of these, 64 (72%) had improved at 3-year follow-up; 56 (63%) had partial, and eight (9%) complete resolution, while 24 (27%) remained unchanged. No relationship between degree of VUR and resolution was observed. The high resolution rate reported by Parvex et al. could not be confirmed in our study. Instead, regression of renal damage was found in only 19% of the children. However, many children with minor renal damage were excluded as they did not have a follow-up DMSA scan. The material described by Parvex et al. might have been of a more benign nature, which is also reflected by a lower percentage of VUR, 36 vs. 63%, in the present study.

Even though renal damage remained unchanged over time in the majority of children, progression occurred in 19%. This is similar to the 11–18% observed in the reflux studies cited above. The risk factors for progression were non-*E. coli* at the index infection, moderate to pronounced renal damage at the index DMSA scan, high-grade VUR and recurrent febrile UTI. However, when performing multivariable regression analysis, only VUR and recurrent UTI remained significant. Furthermore, there was a gender difference where VUR was important for boys and both recurrence and initial damage for girls. Interestingly, there was no relationship between evolution of renal damage and gender, age or degree of inflammation at the index UTI.

This study has some limitations. It is retrospective. The follow-up time is variable, as is the time to investigation. Also there was a substantial number of patients lost to follow-up, but most of these had minor renal damage. Up-take defects found on DMSA scan performed early after acute UTI may be transient. There are different opinions on the optimal time interval between UTI and visualization of permanent renal damage on DMSA scan. While Goldraich et al. recommended an interval of at least 3 months, others have found resolution of defects 6 to 12 months after an acute UTI [9–11, 22]. We used a 90-day interval. There was no significant difference in final renal outcome between DMSA scan performed at 3 to 6 months compared to later after the index UTI. Still, the interval chosen may have influenced the rate of regression. It may be that kidneys under normal resolution of acute damage have been regarded as regression. Speculatively, another explanation could be hypertrophy of surrounding renal parenchyma decreasing the appearance of the isotope up-take defect. As in other studies using DMSA scans for detecting renal damage, it is not possible to differentiate between congenital and acquired damage. However, it is likely that congenital renal damage may also progress.

This study describes deterioration of 4 to 10% in DRF during a follow-up time of 2 to 18 years in 19% of small children with renal damage diagnosed after UTI. The long-term impact of this is unclear. Studies of children with renal damage followed into adulthood have presented conflicting results, where some have shown few complications [12] while others a substantial risk of hypertension [14].

## Conclusions

Most small children with febrile UTI do not develop renal damage and if they do the majority remain unchanged or regress over time. However, up to one-fifth of children with renal damage diagnosed after UTI are at risk of renal deterioration. These children are characterized by the presence of VUR grade III–V and recurrent febrile UTI and may benefit from follow-up.
